# Autophagy promotes MSC-mediated vascularization in cutaneous wound healing via regulation of VEGF secretion

**DOI:** 10.1038/s41419-017-0082-8

**Published:** 2018-01-19

**Authors:** Y. An, W. J. Liu, P. Xue, Y. Ma, L. Q. Zhang, B. Zhu, M. Qi, L. Y. Li, Y. J. Zhang, Q. T. Wang, Y. Jin

**Affiliations:** 1 State Key Laboratory of Military Stomatology & National Clinical Research Center for Oral Diseases & Shaanxi Engineering Research Center for Dental Materials and Advanced Manufacture, Department of Periodontology, School of Stomatology, The Fourth Military Medical University, Xi’an, Shaanxi 710032 China; 2 State Key Laboratory of Military Stomatology & National Clinical Research Center for Oral Diseases & Shaanxi International Joint Research Center for Oral Diseases, Center for Tissue Engineering, School of Stomatology, The Fourth Military Medical University, Xi’an, Shaanxi 710032 China; 3 Xi’an Institute of Tissue Engineering and Regenerative Medicine, Xi’an, Shaanxi 710032 China; 4 Institute of Stomatology, Chinese PLA General Hospital, Beijing, 100853 China; 5 Department of Stomatology, PLA Xizang Military Region General Hospital, Lhasa, Tibet 850007 China

## Abstract

Vascularization deficiency caused a lot of diseases, such as diabetes ulcer and myocardial infarction. Mesenchymal stem cells (MSCs), with the self-renewal and multipotent differentiation capacities, have been used for many diseases treatment through regulation microenvironment. Numerous studies reported that MSCs transplantation could largely improve cutaneous wound healing via paracrine secretion of growth factors. However, whether MSCs take part in the angiogenesis process directly remains elusive. Previous study proved that autophagy inhibited immunosuppressive function of MSCs and prevented the degradation of MSCs function in inflammatory and senescent microenvironment. Here, we proved that autophagy determines the therapeutic effect of MSCs in cutaneous wound healing through promoting endothelial cells angiogenesis and demonstrated that the paracrine of vascular endothelial growth factor (VEGF) in MSCs was required in wound site. We further revealed that autophagy enhanced the VEGF secretion from MSCs through ERK phosphorylation directly. Collectively, we put forward that autophagy mediated paracrine of VEGF plays a central role in MSCs cured cutaneous wound healing and may provide a new therapeutic method for angiogenesis-related diseases.

## Introduction

All types of cutaneous wound healing require de novo angiogenesis for transportation of systematic oxygen, nutrient, and other necessities into proximal wounded sites during the entire tissue repairing process^1^. Mesenchymal stem cells (MSCs) have been considered as a good source of adult stem-like cells and have been used in many clinical applications for their self-renewal, multipotent differentiation capacities, and low immunogenicity^2,3^. Numerous studies suggested that injection of MSCs can effectively treat some diseases such as immune system disease, myocardial infarction (MI), and skin defect^4^. An important aspect of this therapeutic mechanisms of the MSCs have been frequently associated with its paracrine secretion capacity. It has been reported that the hepatocyte growth factor (HGF)-paracrine secreted by injected MSCs promote cardio-protection in MI^5^. Furthermore, a study firstly reported that resveratrol can preserve the paracrine effect of the adipose tissue-derived mesenchymal stem cells (ADMSCs) with senescence on promoting insulin secretion of INS-1 cells^6^. The other study reported that neurotrophin-1 paracrine secreted from MSCs accelerated wound healing in diabetic mice^7^. However, what regulates the paracrine of MSCs in wound healing remains elusive.

Autophagy is a cellular protective process of degrading misfolded proteins and damaged organelles, such as mitochondria and endoplasmic reticulum and maintaining cellular homeostasis under starvation or hypoxia conditions^8–10^. Under inflammatory microenvironment or hypoxic condition, autophagy protected MSCs from apoptosis via AMPK/mTOR pathway^11,12^. Moreover, several studies proved that autophagy inhibited immunosuppressive function of MSCs in autoimmune encephalomyelitis treatment^13^, inhibition of autophagic flux regulated endothelial VWF secretion in angiogenesis^14^. In addition, previous researches also showed that autophagy took part in neurogenesis^15^ and osteogenesis^16^. We previously observed that autophagy prevented the degradation of MSCs function in inflammatory and senescent microenvironment (Unpublished data). However, whether and how autophagy of MSCs controls the therapeutic effect of wound healing is not fully understood.

In this study, we observed that local transplantation of MSCs improved cutaneous wound healing via vascular endothelial growth factor (VEGF)-paracrine secreted from MSCs. Mechanically, we reveal that autophagy in MSCs drives the paracrine secretion of VEGF through directly phosphorylating ERK. These data provide evidence that autophagy determines the therapeutic effect of MSCs in cutaneous wound healing via promoting VEGF secretion by directly phosphorylating ERK.

## Results

### MSCs exhibited higher therapeutic effect on cutaneous wound healing through subcutaneous injection than intravenous injection via promoting local angiogenesis

We have successfully obtained the human MSCs from the bone marrow. MSCs used in this research showed high proliferation rates and multipotent differentiation capacities toward the osteoblast and adipocyte lineages (Supplementary Fig. S1a–c). Meanwhile, MSCs possessed high autophagy level when treated with rapamycin (Supplementary Fig. S1d).

**Fig. 1:**
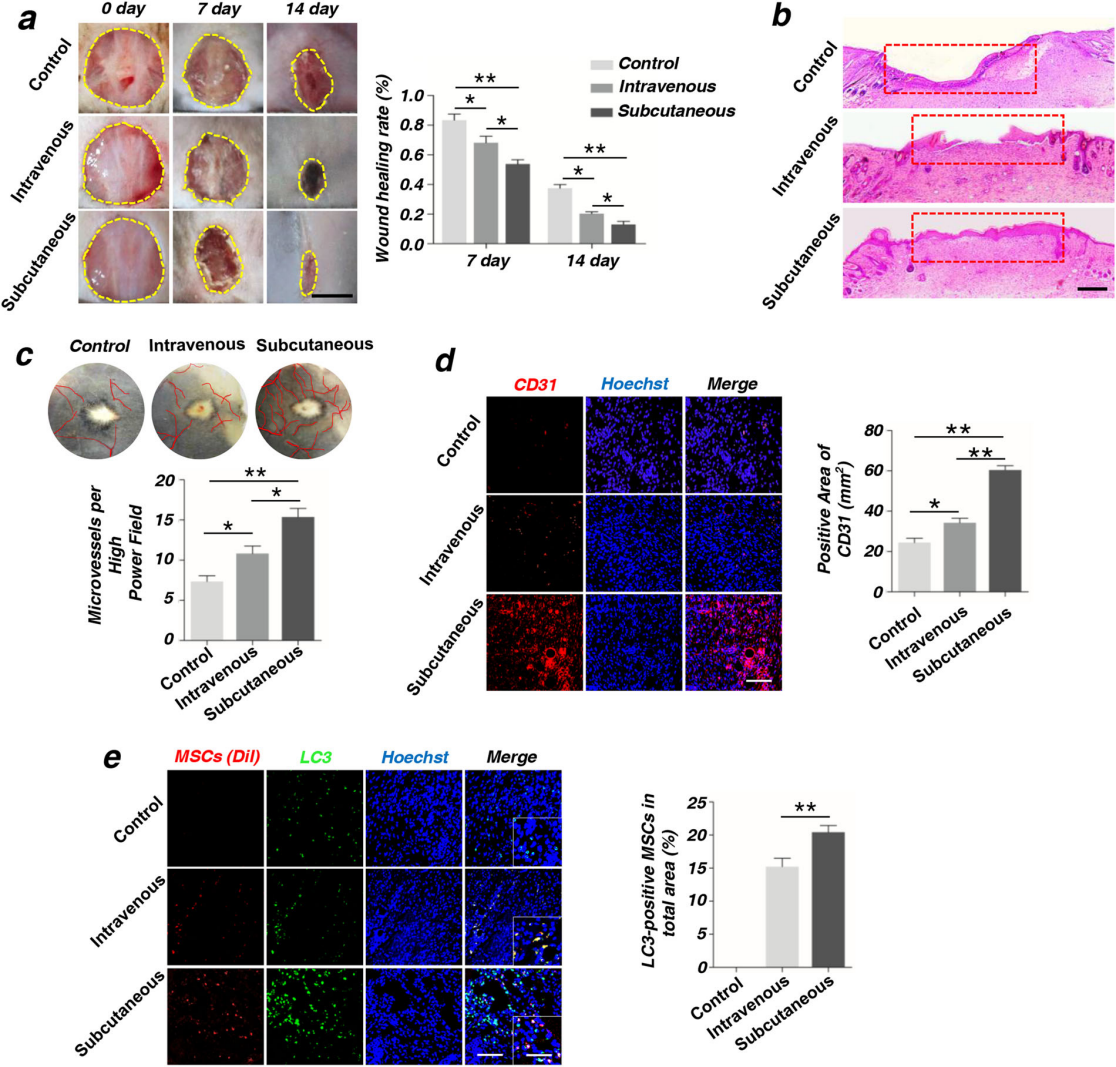
MSCs possess the higher pro-angiogenesis capacity of endothelial cells (ECs) through subcutaneous injection than intravenous injection **a** Images of wound size on the back of mouse and quantification of wound healing rate (%) in different groups. Scale bar = 5 mm. **b** Hematoxylin and eosin (H&E) staining of wound area tissues at 2-week post-operative. The red boxes indicate the healing of wound skin. Scale bar=2 mm. **c** Vascularization state in wound healing skin and quantification of capillary number at 2-week post-operative. **d** Immunofluorescence staining on CD31 of wound area tissues and quantification of positive areas of CD31 at 2-week post-operative. Scale bar = 50 μm. **e** Dio-labeled MSCs (red) and LC3-positive cells (green) at 24 h after injection. The co-stained cells represented the autophagy activation of MSCs, which were then analyzed as the percentage of total detected MSCs (*P < 0.05, **P < 0.01, *n* = 3). Scale bar = 50 μm. The photo in right corner is the locally enlarged image. Scale bar = 10 μm

In a full-thickness skin defects model using C57BL/6 mouse, we firstly compared the wound healing rate of intravenous injection and subcutaneous injection of MSCs by quantitatively measuring the skin healing rate (Fig. 1a). We found that subcutaneous injection was much more efficient than intravenous injection in skin wound healing. Moreover, histological analysis showed that MSCs injected through subcutaneous promoted the regeneration of epithelium at post-operative 14d (Fig. 1b). Vascularization was an essence to wound healing and tissue repairing^17^. Further the blood vessels and capillary net in the wound area was observed by Integrated microscope. Our results showed that more capillaries were generated in subcutaneous injection than intravenous injection (Fig. 1c). Moreover, immunohistochemistry staining of an angiogenesis marker, CD31 in the wound area indicated that, the subcutaneous injection group had more positive points of CD31, compared with the intravenous injection group. Quantification analyses of positive areas of CD31 were statistically significant at 2-week post-operative (Fig. 1d). Next, after 24 h post injection, the surviving MSCs could be observed around the wound area by immunofluorescence staining. Meanwhile, immunofluorescence staining showed that the subcutaneous injection group expressed much more LC3 compared with intravenous injection group, and the percentage of LC3-positive MSCs had statistical significance (Fig. 1e). We further observed the Dio-labeled MSCs (red) and LC3-positive cells (green) at 48 h after subcutaneous injection (Supplementary Fig. S2). The co-stained MSCs represented the autophagy activation. Therefore, the subcutaneous injection was used in the subsequent experiments.

### Autophagy determines the therapeutic function of MSCs in wound healing via promoting the angiogenesis

**Fig. 2:**
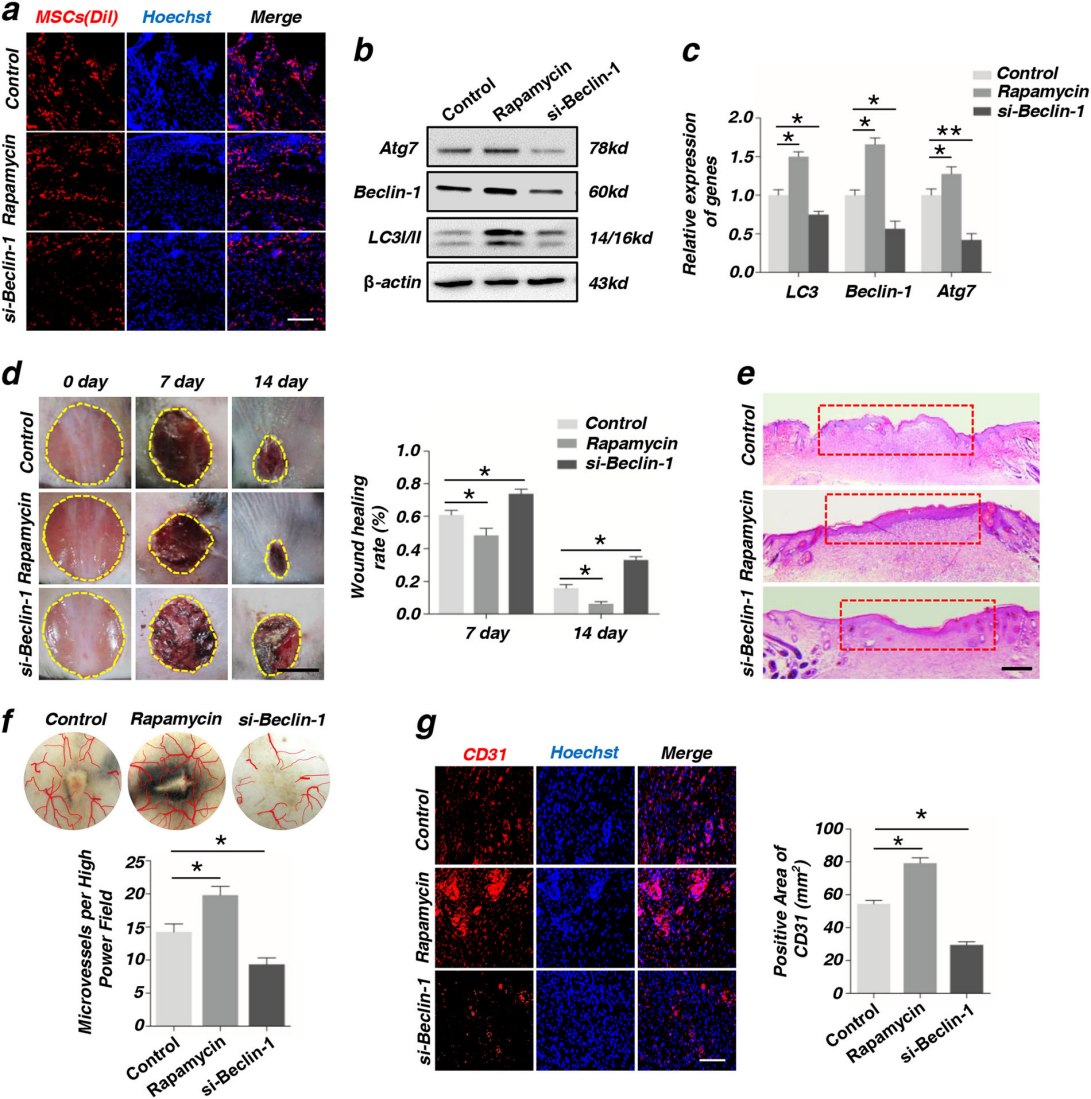
Autophagy enhances the pro-angiogenesis capacity of endothelial cells (ECs) after culturing with MSCs via promoting angiogenesis in injury part **a** Dio-labeled MSCs (red) were detected by immunofluorescence assay at 24 h after injection. Scale bar = 50 μm. **b** The proteins of Atg7, Beclin-1, and LC3I/II of MSCs treated with rapamycin and si-Beclin-1 were tested by western blot. **c** The genes of Atg7, Beclin-1, and LC3 of MSCs treated with rapamycin and si-Beclin-1 were tested by RT-PCR. **d** Images of wound size on the back of mouse and quantification of wound healing rate (%) in different groups. Scale bar = 5 mm. **e** Hematoxylin and eosin (H&E) staining of wound area tissues at 2-week post-operative. The red boxes indicate the healing of wound skin. Scale bar = 2 mm. **f** Vascularization state in wound healing skin and quantification of capillary number at 2-week post-operative. **g** Immunofluorescence staining on CD31 of wound area tissues and quantification of positive areas of CD31 at 2-week post-operative (*P < 0.05, **P < 0.01, *n* = 3). Scale bar = 50 μm

As before, we found that autophagy of MSCs was associated with angiogenesis. To further illustrate the relationship between autophagy and angiogenesis, we changed the autophagy level in MSCs with rapamycin or si-Beclin-1, as Beclin-1 is essential in initiation and maturation of autophagosomes. First, we compared the “control” (MSCs without any treatment) group with MSCs transfected with “si-control” group, the results of western blot, immunofluorescence staining of CD31 and wound healing rates indicated that there were no differences between two groups (Supplementary Fig. S3). Therefore, we used one “control” group (MSCs without any treatment) in the subsequent experiments. Before injection, MSCs treated with rapamycin or si-Beclin-1 were labeled with red “Dio”. After 24 h (Fig. 2a) and 48 h (Supplementary Fig. S4) post injection, the Dio-labeled MSCs (red) in different groups were observed around the wound area by immunofluorescence staining. Meanwhile, the western blot data showed that the expression of Atg7, Beclin-1, and LC3I/II were much higher in rapamycin group than in si-Beclin-1 group (Fig. 2b). The reverse transcription-polymerase chain reaction (RT-PCR) arrived the same conclusion (Fig. 2c). All the primer sequences were listed in Supplement S1. The rapamycin group had a better wound healing through local injection MSCs at post-operative day 7 and 14, compared with control group. On the other hand, the si-Beclin-1 group reduced the skin healing. At the same time, wound healing rate, which was used to quantitatively evaluate skin healing, was significantly increased in rapamycin group and decreased in si-Beclin-1 group (Fig. 2d). Moreover, histological analysis showed that MSCs treated with rapamycin promoted the regeneration of epithelium at post-operative day 14 (Fig. 2e). Further, the blood vessels and capillary net in the wound area was observed by integrated microscope. The results indicated that more capillaries were generated in rapamycin group than control group (Fig. 2f). Besides, the results of immunofluorescence staining showed that rapamycin group had more and si-Beclin-1 group had less positive CD31 than control group. Further statistical analysis of positive CD31 indicated that the differences between each group were statistical significant (Fig. 2g).

### Autophagy determines the angiogenesis of endothelial cells (ECs) through co-culturing with MSCs

**Fig. 3:**
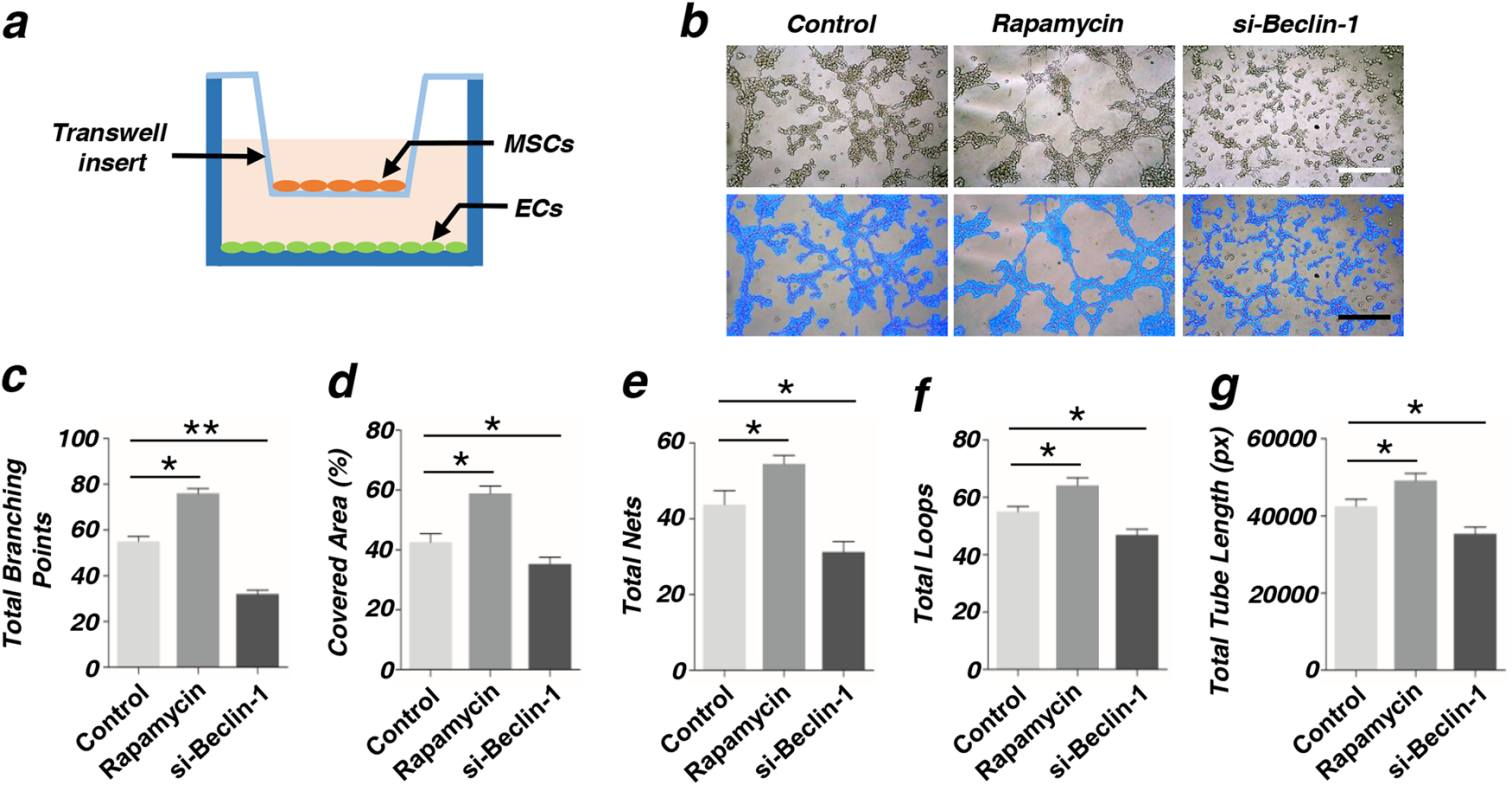
Autophagy determines the angiogenesis of endothelial cells (ECs) after being co-cultured with MSCs **a** Diagram of co-culture system using a Transwell insert. ECs were co-cultured with MSCs (pretreatment with rapamycin or si-Beclin-1) in a Transwell system, and ECs were then collected for further analysis. **b** In vitro tube formation of ECs at 6 h. Scale bar = 20 μm. **c**–**g** Quantification of total branching points, covered areas, total nets, total loops, and total tube length were measured by analytical software Image-Pro Plus 6.0 (IPP) (*P < 0.05, **P < 0.01, *n* = 3)

**Fig. 4:**
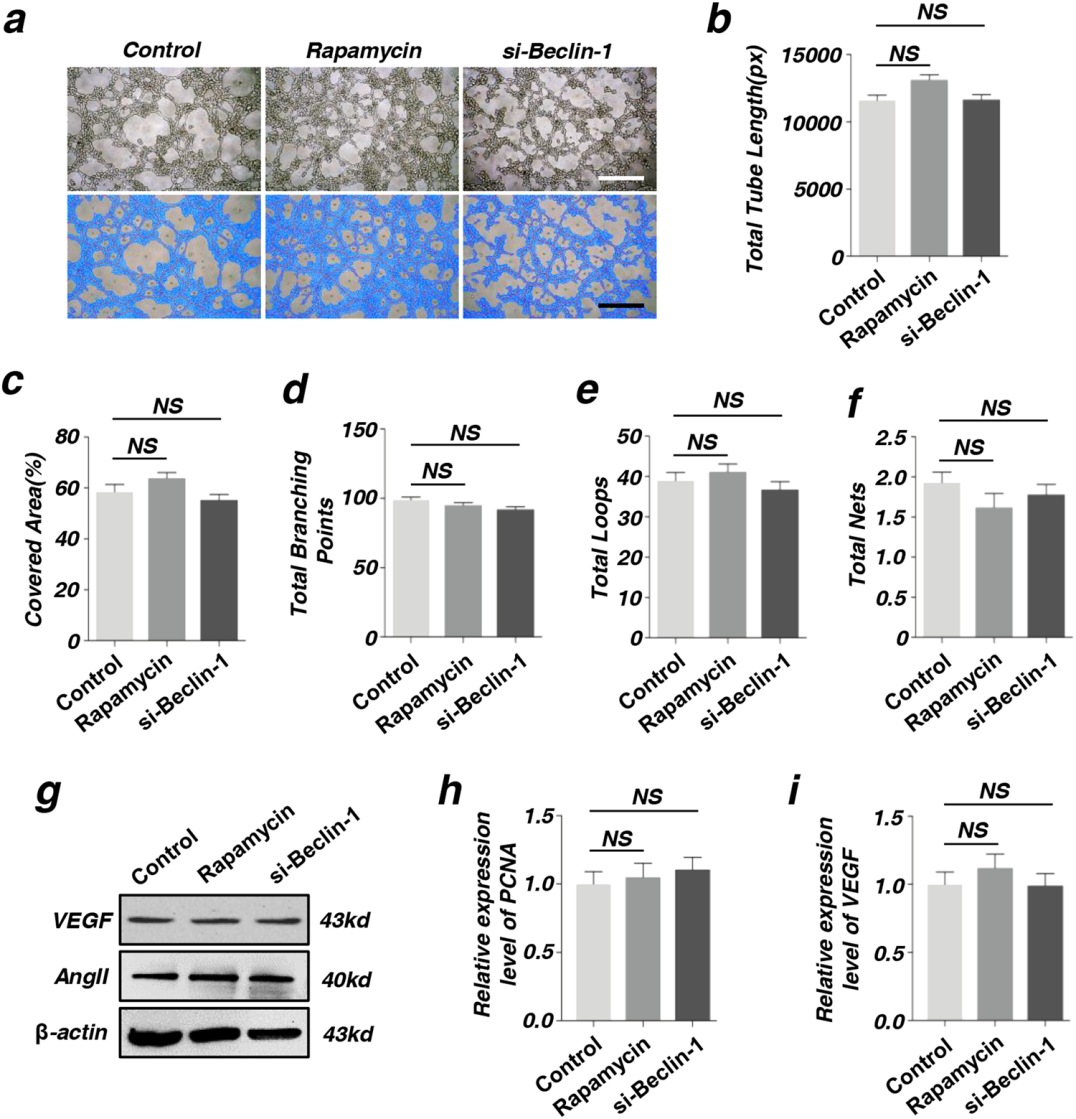
Autophagy had no direct effect on the angiogenesis of endothelial cells (ECs) **a** In vitro tube formation of ECs treated with rapamycin or si-Beclin-1 at 6 h. Scale bar = 20 μm. **b**–**f** In vitro tube formation of EPCs at 6 h and quantification of total tube length, covered areas, total branching points, total loops, and total nets were measured by analytical software Image-Pro Plus 6.0 (IPP). **g** The expression of angiogenesis-related proteins VEGF and AngII of ECs were detected by western blot. **h**, **i** The expression of angiogenesis-related genes PCNA and VEGF of ECs were detected by RT-PCR. (^NS^*P* > 0.05, *n* = 3)

We successfully established a co-culture system (Fig. 3a) and tested the tube formation of ECs after being co-cultured with MSCs at 6 h. Complete tubes were formed in rapamycin group and the cells were dispersedly arranged in si-Beclin-1 group (Fig. 3b). Total tube length, total branching points, covered areas, total nets and total loops were measured using analytical software Image-Pro Plus 6.0 (IPP). Our data showed that the differences between the two groups were statistical significant (Fig. 3c–g). For avoiding the effect of autophagy on the angiogenesis of ECs, we measured the tube formation of ECs after being treated with rapamycin or si-Beclin-1. The data showed that there was no statistical significance between two groups (Fig. 4a–f). Further, we measured the protein expression level of VEGF and AngII (Fig. 4g), RNA expression level of VEGF and PCNA (Fig. 4h-i), These results indicated that autophagy had no direct effect on the angiogenesis of ECs.

Besides, rapamycin, as a immuno-suppressor, was used to treat immune system diseases in many previous reports. For finding out whether local application of rapamycin could promote the skin wound healing, we observed the effect of rapamycin on local application. The skin wound healing rate and wound healing result had no statistical differences (Supplementary Fig. S5a). Histological analysis showed that rapamycin did not affect the epithelial regeneration (Supplementary Fig. S5b). Further immunohistochemistry of CD31 showed that there were no significant differences on positive of CD31 signals between two groups (Supplementary Fig. S5c).

### Autophagy promotes VEGF secretion of MSCs directly through phosphorylating ERK1/2

**Fig. 5:**
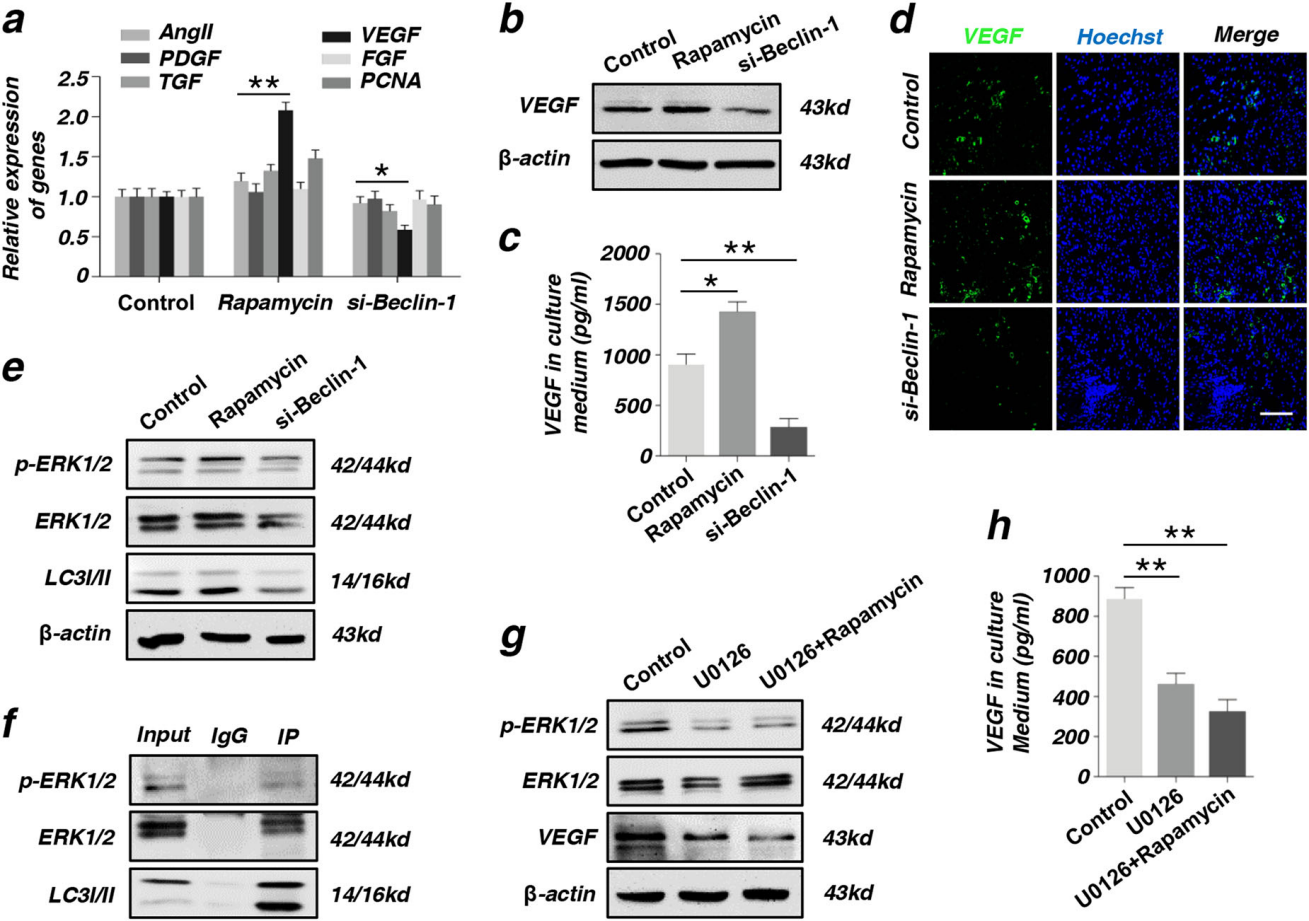
Autophagy promote the secretion of VEGF through phosphorylating ERK1/2 **a** Screening angiogenesis-related genes (VEGF, AngII, PDGF, FGF, TGF, and PCNA) of MSCs after being treated with rapamycin or si-Beclin-1 by RT-PCR. **b** The expression of angiogenesis-related proteins VEGF in MSCs as detected by western blot. **c** The secretion of VEGF in culture medium of MSCs treated with rapamycin or si-Beclin-1 was detected by ELISA assay. **d** Immunofluorescence staining on VEGF of wound area tissues at 2-week post-operative. Scale bar = 50 μm. **e** The detection of protein LC3I/II, ERK, and p-ERK in MSCs after being treated with rapamycin or si-Beclin-1 by western blot. **f** LC3 interacts with ERK in vivo. Immunoblots showing co-immunoprecipitation of LC3 with ERK. **g** The expression of VEGF was reduced in MSCs treated with ERK inhibitor U0126 by western blot. **h** The secretion of VEGF was reduced in culture medium of MSCs treated with ERK inhibitor U0126 by ELISA assay (*P < 0.05, **P < 0.01, *n* = 3)

VEGF, as one of the numerous angiogenesis-related factors, played a vital role in angiogenesis during wound healing process^18^. In this study, we found that the RNA expression level of VEGF is positively correlated with autophagy by RT-PCR (Fig. 5a). Further, when autophagy was activated, the protein expression level of VEGF was also increased (Fig. 5b). Meanwhile, ELISA assay data showed that the secretion of VEGF in cultured medium was also higher in rapamycin group (Fig. 5c). Immunofluorescence staining confirmed the positive relation between VEGF and wound healing. As shown in Fig. 5d, the expression of VEGF increased in rapamycin group and decreased in si-Beclin-1 group, compared with control group.

As previous paper reported, increased nuclear LC3-II punca would activate the ERK pathway^19^. p-ERK pathway plays an important role in the paracrine secretion of VEGF^20^. Then we upregulated the autophagy level in MSCs with rapamycin and downregulated the autophagy level in MSCs through transfected si-Beclin-1. We measured the protein expression level of VEGF and p-ERK pathway. We showed that the p-ERK pathway was activated by rapamycin and expression of VEGF also increased. At the same time, the expression of VEGF was reduced when p-ERK pathway was inhibited by transfection of si-Beclin-1. In brief, p-ERK pathway upregulated the paracrine of VEGF in MSCs (Fig. 5b, e). For verifying interactions between p-ERK and LC3, we co-immunoprecipitated LC3 from total proteins from MSCs and analyzed pull-downs for p-ERK cascade components. Indeed, the results revealed that LC3 co-immunoprecipitated with ERK1/2. In addition, p-ERK1/2 were also detected in pull-downs of LC3 from total proteins (Fig. 5f). To further analyze the role of p-ERK pathway in the paracrine effect of VEGF in MSCs, we used the ERK pathway inhibitor U0126 to treat MSCs and then detected the protein expression level of VEGF in MSCs by western blot. The expression of VEGF significantly reduced in MSCs after treated with U0126 (Fig. 5g). Meanwhile, ELISA data showed that the secretion of VEGF in the culture medium also reduced in U0126 group (Fig. 5h). However, activating the autophagy of MSCs after inhibiting the p-ERK pathway, the protein expression level of VEGF and the secretion of VEGF did not increase.

### VEGF is crucial in cutaneous wound healing via promoting the angiogenesis of ECs

**Fig. 6:**
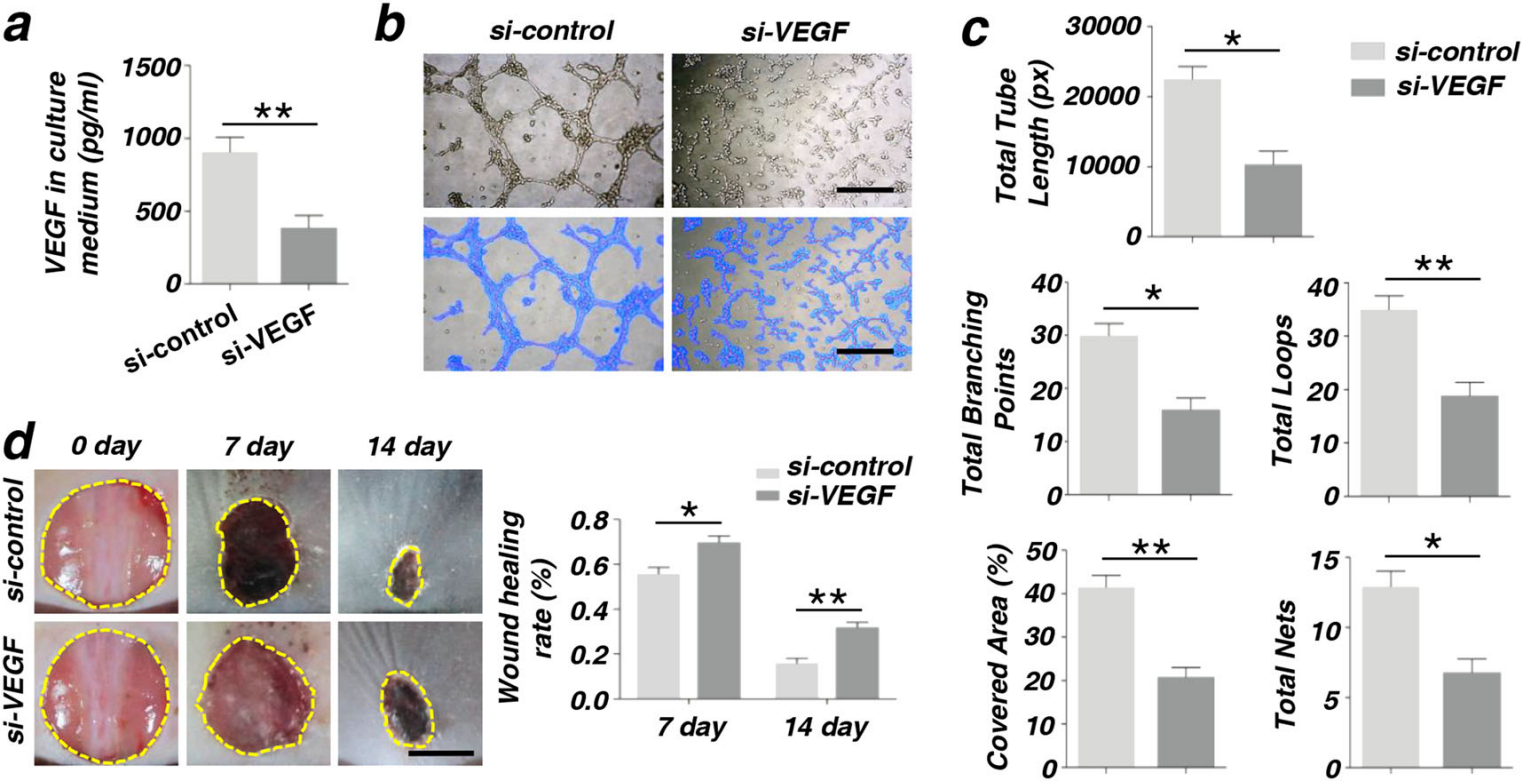
The angiogenesis capacity of endothelial cells (ECs) was decreased and the wound healing rate was reduced in MSCs after being treated with si-VEGF **a** The secretion of VEGF was reduced in culture medium of MSCs treated with si-VEGF by ELISA assay. **b**, **c** In vitro tube formation of EPCs at 6 h and quantification of total tube length, total branching points, covered areas, total nets, and total loops were measured by analytical software Image-Pro Plus 6.0 (IPP). Scale bar = 20 μm. **d** Images of wound size on the back of mouse and quantification of wound healing rate (%) (*P < 0.05, **P < 0.01, *n* = 3). Scale bar = 5 mm

Consistent with previous reports, we confirmed the importance of VEGF in angiogenesis. We knocked down the gene VEGF by transfecting si-VEGF, and tested the transfection efficiency by western blot and RT-PCR (Supplementary Fig. S6). The data showed that both the RNA and protein expression levels of VEGF efficiently decreased 48 h after transfection. The secreted VEGF in the culture medium dramatically decreased in MSCs after transfecting with si-VEGF (Fig. 6a). Moreover, after being co-cultured with MSCs transfected with si-VEGF, the ability of tube formation was also down in ECs (Fig. 6b). Total tube length, total branching points, covered areas, total nets, and total loops were measured by analytical software Image-Pro Plus 6.0 (IPP). We showed that there were no significant differences between two groups (Fig. 6c). The in vitro experiments the wound healing rate was quantitatively measured and indicated that MSCs transfected with si-VEGF inhibited skin wound healing (Fig. 6d).

### Autophagy promotes the therapeutic effect of MSCs via VEGF secretion

**Fig. 7:**
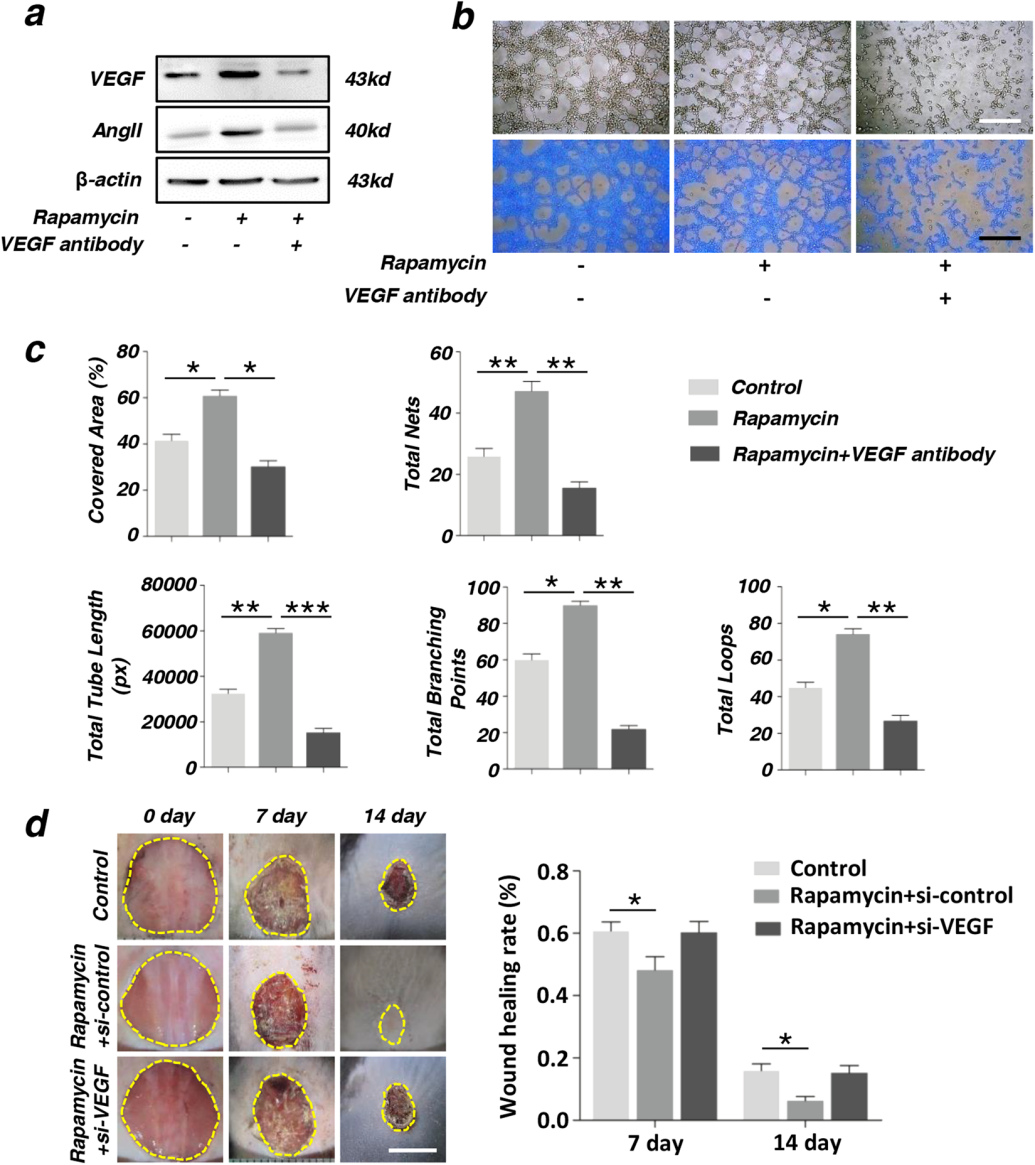
The secreted VEGF from MSCs played a critical importance in the process of angiogenesis of endothelial cells (ECs) **a** expression of angiogenesis-related proteins VEGF and AngII in ECs were detected by western blot. **b**, **c** In vitro tube formation of ECs at 6 h and quantification of total tube length, total branching points, covered areas, total nets, and total loops were measured by analytical software Image-Pro Plus 6.0 (IPP). Scale bar = 20 μm. **d** Images of wound size on the back of mouse and quantification of wound healing rate (%) (*P < 0.05, **P < 0.01, *n* = 3). Scale bar = 5 mm

To analyze whether the secretion of VEGF played an important role in the angiogenesis of ECs, we used the VEGF antibody to neutralize VEGF secreted from MSCs in the culture medium when co-cultured with ECs. Then ECs were collected for western blot and tube formation experiment. Western blot data showed that protein expression level of VEGF and AngII increased in rapamycin group and reduced in VEGF antibody group, compared with the control group (Fig. 7a). Many tubes were formed in rapamycin group and the number of tubes reduced when VEGF antibody were added to the culture medium (Fig. 7b). Similarly, total tube length, total branching points, covered areas, total nets, and total loops were measured by analytical software Image-Pro Plus 6.0 (IPP). These data showed that the two groups were significantly different (Fig. 7c). Further in vitro experiment indicated that upon activation of autophagy, treatment with si-VEGF would reduce the wound healing efficiency compared with the rapamycin + si-control group and the difference of wound healing rate has statistical significance (Fig. 7d).

## Discussion

In the current study, we found that autophagy plays an important role on VEGF-mediated angiogenesis of ECs. Formation of new blood vessels, which mediates the transport of nutrients, oxygen, growth factors and circulating cells, is an essential process for skin wound healing and tissue regeneration^21,22^. Local injection of MSCs enhanced angiogenesis in skin wound healing and thus promoted wound healing. At the same time, we found that autophagy also promoted the process of skin wound healing. Autocrine VEGF maintains vascular homeostasis through regulation of autophagy level, thereby ensuring physiological metabolism and EC survival^23^.

To confirm that autophagy plays an important role in angiogenesis, we interfered with autophagy in MSCs with rapamycin or si-Beclin-1 before injection. In vivo experiment showed that increased autophagy of MSCs accelerated angiogenesis in skin wound healing and thus promoted wound healing. In vitro co-culture system showed that MSCs enhanced the ability of angiogenesis of ECs. Vascularization of ECs were enhanced when being co-cultured with MSCs treated with rapamycin. Previous paper reported that autophagy would directly affect the angiogenesis of ECs^24^, but in our study, interfering autophagy did not affect the angiogenesis of ECs. The reason for these different results is possibly that the ECs used in the studies were different. In our study we used ECs line and in the previous paper they used primary cultured cells.

Autophagy is a cellular protective behavior of degrading misfolded proteins and damaged organelle in order that providing essential elements for maintaining cell metabolism under starvation or hypoxia conditions, with close connections to human physiology and disease^8–10^. Autophagy enhanced survival of ECs by inhibiting apoptosis under hypoxia condition and promotes proliferation and differentiation of ECs, which is important in angiogenesis during the regeneration of the ischemic tissue^25^. Rapamycin, as a immuno-suppressor, was reported to treat immune system diseases. Previous paper reported that rapamycin promoted lifespan and delayed age-related diseases through inhibition of the mechanistic target of rapamycin (mTOR) signaling pathway in model organisms including mice^26^. Meanwhile, rapamycin was used as an activator of autophagy. But in this study, local apply rapamycin had no effect on the skin wound healing (Supplementary Fig. S5). Instead, local injection of MSCs treated with rapamycin promoted skin wound healing.

Injection of MSCs can accelerate angiogenesis thus the wound healing process by secreting numbers of growth factors such as VEGF, platelet-derived growth factor (PDGF), fibroblast growth factor (FGF), transforming growth factor-β (TGF-β) and several integrin^27^. Among these factors, VEGF is a secreted mitogen and associates with angiogenesis by stimulating migration, proliferation, and tube formation of ECs primarily through binding to the VEGFR1 or VEGFR2^28,29^. Although the underlying mechanism of MSCs-based therapy remains unclear, VEGF paracrine secreted from MSCs has been reported to have a central role in wound healing^30^. VEGF promotes ECs into blood vessel termed as vasculogenesis^31,32^. Previous paper reported that autophagy induction or inhibition was closely correlated with VEGF-mediated angiogenesis^33^. In this study, we found that the expression level of VEGF increased along with the activating level of autophagy in MSCs. Additionally, interfering with autophagy affected VEGF-induced angiogenesis.

**Fig. 8:**
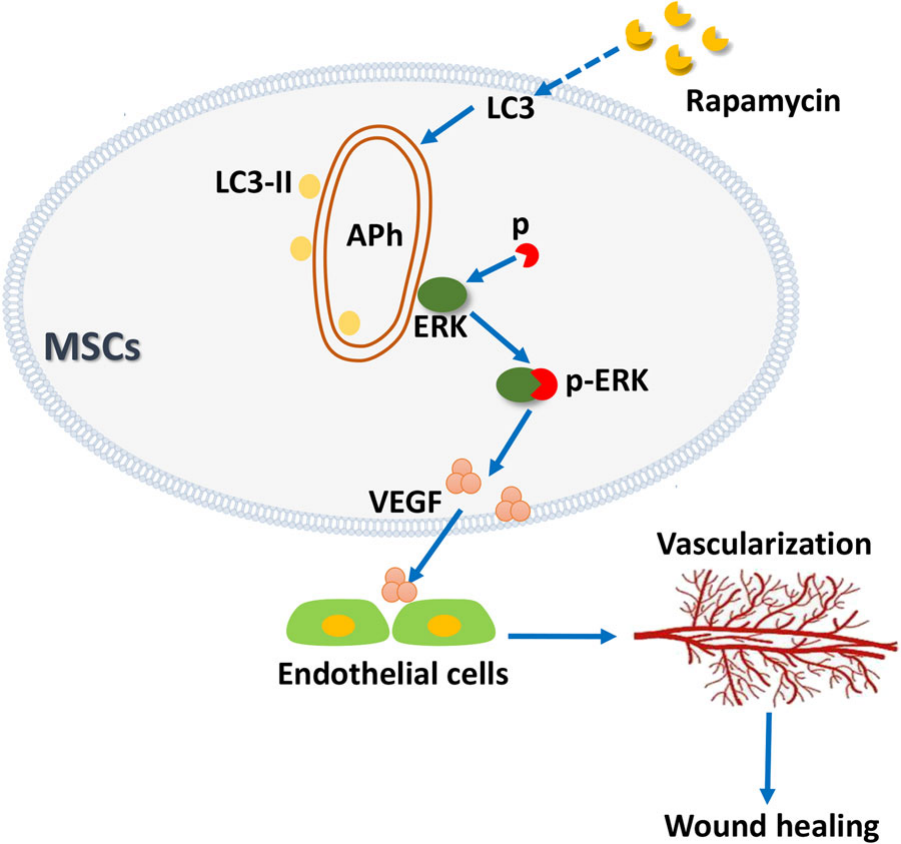
Autophagy increase the VEGF secretion from MSCs through regulating ERK phosphorylation, and VEGF further promote vascularization of endothelial cells

Recent studies indicated that the therapeutic effects of MSCs injection that happened via a paracrine mechanism. Exosome, a most important paracrine trafficking mechanism of MSCs, can be directly used to enhance skin wound healing by promoting angiogenesis^34^. ERK, an ubiquitose kinase, has many functions in MSCs^35^. ERK has been shown to be an important directly modulator in VEGF expression in fibroblasts^36^, especially under hypoxia condition^37^. In this study, we have reported that rapamycin pretreatment stimulated the paracrine of VEGF in MSCs through the ERK1/2 pathway. Furthermore, our study confirmed that autophagy increases VEGF production, an angiogenesis factor, by activation of the ERK signal pathway as illustrated in Fig. 8.

In summary, the present study proved the efficiency of MSCs treated with rapamycin aggregate in facilitating full-layer cutaneous wound healing and regeneration. Furthermore, we demonstrated that autophagy promoted the paracrine secretion of VEGF in MSCs. The VEGF accelerated the angiogenesis of ECs. These findings provided insights into the novel role of autophagy as a central coordinator to regulate the therapeutic effect of MSCs.

## Materials and methods

### Isolation and culture of human MSCs

The experimental protocols were approved by the committee of ethics (Institutional Review Board for Human Subjects Research) of the Fourth Military Medical University (FMMU, Xi’an, China) and informed consent was obtained from each subject. The healthy bone marrow was harvested from the department of hematology, Xijing Hospital of FMMU. All samples were used for cell culture within 2 h separation.

Human primary MSCs were purified from heparinized bone marrow samples of adult donors (ages from 24–30) and cultured as previously described in our laboratory^38^. Briefly, the mononuclear cells were obtained by ficoll density gradient centrifugation, suspended in regular culture medium consisting of α-MEM (Invitrogen, Carlsbad, CA) supplemented with 10% fetal bovine serum, 0.292 mg/ml glutamine, 100 U/ml penicillin G, 100 μg/ml streptomycin (all from Invitrogen), and seeded in 10-cm-diameter culture flasks (Costar, Cambridge, MA) at a density of 1 × 10^5^ cells/ml. Next day, the culture supernatants were replaced by fresh medium to allow expansion of the adherent cell fraction. Cells at passage 3 were used for all experiments.

### Endothelia progenitor cells culture

Endothelia progenitor cell line, was purchased from the American Type Culture Collection (Rockville, MD, USA). For regular (nonpolarized) culture, the cells were grown in α-MEM supplemented with 10% fetal bovine serum (FBS), and antibiotics streptomycin/penicillin G (Sigma-Aldrich).

### Characterization of MSCs

#### Colony-forming unit assays

The human MSCs (P_3_) suspended in α-MEM were plated at a density of 0.5 to 2 × 10^3^ in 10-cm-diameter culture dishes (Corning, Lowell, MA, USA) and cultured in complete medium for colony-forming unit-fibroblast (CFU-F) assays. All cultures were fixed on day 14 with 4% paraformaldehyde and stained with 1% toluidine blue. After washing three times with distilled water, the number of colonies was counted for contrastive analysis. For colony number counting, only aggregates of 50 or more cells viewed under the microscope were scored as colonies. The experiment was repeated at least three times.

#### Cell proliferation assays

The cell proliferative potential was determined by the 3-(4,5-dimethylthiazol-2-yl)-2,5-diphenyltetrazolium bromide (MTT) assay. Briefly, the MSCs (P_3_) were plated at a density of 2 × 10^3^ cells/well into 96-well plates (Corning) and cultured with 200 μl α-MEM, then the cells incubated at 37 °C in a humidified atmosphere containing 5% CO_2_ for cell attachment and spreading. At time points of day 1 to 8, 20 μl MTT solution (Sigma-Aldrich, 5 mg/ml) was added to each well and incubated at 37 °C for 4 h. Then the medium was discarded, and formazan salts were dissolved with 150 ml dimethylsulfoxide (DMSO, Sigmae Aldrich). The absorbance in individual wells was read at 540 nm by a microplate reader (ELx800, BioTek Instruments Inc., USA) and expressed as optical density with SoftMax Pro software.

#### Osteogenic differentiation assays

The detection of multiple differentiation capacities of MSCs were conducted according to the methods previously described^39^. Briefly, a total of 2 × 10^5^ MSCs (P_3_) with α-MEM were seeded into each well of a 6-well plate. Until 80% confluence, the medium was changed to osteogenic medium containing 10% FBS, 0.292 mg/ml glutamine, 100 units/ml penicillin, 100 mg/ml streptomycin, 5mM l-glycerophosphate (Sigma-Aldrich), 100 nM dexamethasone (Sigma-Aldrich), and 5 mg/ml ascorbic acid. The induction medium was changed every 2 days. After 4 weeks culture, the cells were washed twice in PBS and fixed with 4% paraformaldehyde and then stained with 1% alizarin red solution (Sigma-Aldrich) in Tris–HCl (pH 8.3). After being washed twice with distilled water, and stained calcium nodules were identified microscopically.

#### Adipogenic differentiation assays

Briefly, a total of 2 × 10^5^ MSCs (P_3_) with α-MEM were seeded into each well of a six-well plate. Until 80% confluence, the medium was changed to adipogenic medium containing 10% FBS, 0.292 mg/ml glutamine, 100 units/ml penicillin, 100 mg/ml streptomycin, 2 mM insulin (Sigma-Aldrich), 0.5 mM isobutyl-methylxanthine (Sigma-Aldrich), and 10 nM dexamethasone (Sigma-Aldrich). The induction medium was changed every two days. After 3 weeks culture, the cells were washed twice in PBS and fixed with 4% paraformaldehyde and then stained with 0.3% Oil Red O (Sigma-Aldrich) solution. After being washed twice with distilled water, and lipid droplets were observed and photographed under a phase-contrast inverted microscope.

### Co-culturing

For purpose of investigating angiogenesis of ECs in vitro, MSCs were treated with rapamycin (100 nM) or si-Beclin-1 then co-cultured with ECs using Transwell filters (12 mm internal diameter, 0.4 μm pore size) (Corning, Incorporation, NY, USA). The MSCs (about 2 × 10^5^cells/well) were cultured on Transwell (up chamber) and ECs (approximately 1.65 × 10^5^cells/well) were seeded on the bottom chamber of the culturing well in α-MEM with 10% FBS, according to different experiments. After co-culturing, the ECs were collected for subsequent experiments.

### Full-thickness cutaneous wound model

All the animal experiments in this study were conducted according to the committee guidelines of the Fourth Military Medical University (FMMU) for animal experiments, which met the NIH guidelines for the care and use of laboratory animals. A total of forty 8-week-old C57BL female mice used in this study were acquired from the experimental animal center of FMMU. The full-thickness cutaneous wound model was established according previously described^40^. Briefly, the mice were anesthetized by 5% pelltobarbitalum natricum (25 mg/kg) and Sumianxin (0.1 ml/kg), and a 1-cm diameter circumference was created by punch biopsy instrument with moderate force on the back of the mouse. Next, the middle of the outlined region of skin was sharply excised along the outline with a pair of scissors. The excised tissues were full-thickness skin in depth, leaving subcutaneous dorsal muscle exposed after excision. The excised tissues were full-thickness skins in depth, and the dorsal subcutaneous muscle was exposed after excision.

Before injecting, MSCs were treated with rapamycin or si-Beclin-1 for 3 days, 2 × 10^6^ cells were suspended in 4 ml of phosphate-buffered saline and labeled with “Dio” labeling solution in accordance with the manufacturer’s instructions, then the cells were intradermally injected around each wound or intravenous injection. MSCs (4 ml) without any treatment were used as control. Using the subcutaneous injection, mice were randomly divided into six groups (eight animals in each group): Control, Rapamycin, Si-beclin-1, Si-VEGF, Rapamycin + si-control, Rapamycin + si- Beclin-1.

### Wound healing assay

In order to evaluate the rate of wound healing in wound area, the wound was photographed by camera after surgery at time points of day 0, 7, and 14. Then the photos were uploaded to an appropriate computer platform and were analyzed using the Image J image analysis software (NIH image software). All the photos were taken with the experimental mouse placed adjacent to a metric ruler that was used for distance calibration and standardization, and the wound area was calculated allowing subsequent statistical analysis. The percentage of wound closure was calculated as follows: area of actual wound/area of original wound × 100%.

### Vascularization of healing wound surface

At day 14 after operation, the mice were euthanized and a full-thickness cutaneous tissue of wound healing and adjacent tissue were obtained. Tissue samples were carefully placed on the bottom of a 10-cm-diameter culture dish and spread with moderate tension to make it unfolded. Next, the vascular infiltration of wound bed biopsy was immediately observed when standard incandescent illumination was directed into the dish under the specimen, then photos were taken with a camera. After visualization of the forming vessels, skin samples were divided into two parts. One part was fixed with 4% paraformaldehyde and prepared for the following H&E and immunohistochemistry staining. The other was dehydrated by 30% sucrose and then embedded in optimum cutting temperature (Leica) for immunofluorescent analysis.

### Tube formation assay

Tube formation assay was carried out as previously described^41^ with a minor modification in vitro. Briefly, 50 μl matrigel (BD Biosciences, San Jose, CA) was added to each well of a 96-well plate and then allowed to be polymerized at 37 °C for half an hour. After co-culture with MSCs, ECs were seeded at density of 2 × 10^4^ cells/well and incubated for 4–6 h. Obvious tube formation was observed under a phase-contrast microscope and take photos incidentally. Then the photos were uploaded to the system of *Image Analysis Platform of Wimasis* for analysis. The photos of blue staining were obtained from this software. Meanwhile, we got the data of the covered areas, the number of branch points, tube length, total loops, and total nets in three randomly selected fields from three independent experiments. Then the tube formation was measured by analyzing this data.

### H&E staining

The healing skin (5 mm^2^) were removed by scissors and fixed in 4% paraformaldehyde at 4 °C overnight. Then the obtained specimens gradually were dehydrated, embedded in paraffin blocks and cut into 4-μm-thickness sections. Sections were subsequently subjected to H&E staining and take photos by light microscopy.

### Immunohistochemistry staining

To quantify the vascular formation in wound healing, the expression of CD31 was detected by immunohistochemistry. As before, the obtained samples were fixed with 4% paraformaldehyde at 4 °C overnight and embedded in paraffin blocks. Then the 4-μm-thickness sections were prepared for immunohistochemistry. Immunohistochemical staining was performed as previously described^42^. The tissue sections were de-waxed in xylene and dehydrated using graded ethanol. 0.3% trypsin (Sigma-Aldrich) was used for antigen retrieval and endogenous peroxidase was blocked using 3% H_2_O_2_ at room temperature for 30 m. Then the sections were blocked with 15% goat serum at 37 °C for 30 m, incubated with primary antibodies CD31 (Abcam,1:200 dilution) at 4 °C overnight, incubated with second antibody at 37 °C for 1 h. Slides were then incubated in avidin-biotin complex (SABC) (Boster) at 37 °C for 20 m and diaminobenzidine (DAB) for 5 m. Finally, the positive areas were observed under an inverted microscope (BX-51, Olympus, Japan).

### Immunofluorescence staining

“Dio” labeled MSCs in vitro were injected into the subcutaneous of skin. The wound area tissues obtained were used for detection of Dio-positive cells infiltration after 48 h and CD31- and VEGF-positive cells infiltration after 2 weeks, respectively. Frozen sections were prepared and the slides were incubated with anti-CD31 primary antibody and anti-VEGF (all from Santa Cruz Biotech, Santa Cruz, CA) primary antibody at 4 °C for overnight. Next day, the slides were then incubated with Alexa Fluor 488 goat anti-rabbit IgG secondary antibody (Invitrogen, Carlsbad, CA) at 37 °C for 2 h. The cell nucleus were stained with Hoechst 33342 dye. The signals were examined by a laser scanning confocal microscope (IX71; Olympus, Tokyo, Japan). In each section, three high-power fields in wound area were randomly selected and photographed.

### Transfection of siRNA

According to the manufacturer’s instructions of siRNA transfection, cells were cultured in 6-well plates and fresh medium without FBS was changed 30 m before preparing for transfection. Then the cells were transiently transfected with 100 pmol si-Beclin-1 or si-VEGF (Santa Cruz Biotech, Santa Cruz, CA) using Lipofectamine 2000. Six hours later, the medium was changed to complete medium (α-MEM with 10% FBS). For real-time PCR assay, total cellular RNA was isolated 24 h after siRNA transfection. For detection of VEGF protein secretion levels, the culture medium was collected for ELISA at 24 h and total cellular protein was extracted at 72 h after siRNA transfection.

### RNA isolation and real-time PCR

Total cellular relative mRNA was isolated from cultured cells using TRIzol reagent (Invitrogen Life Technology, Carlsbad, CA), according to the manufacturer’s instructions. The first-strand cDNA synthesis was performed according to the manufacturer’s instruction (SuperScript III; Invitrogen, Carlsbad, CA). Real-time PCR was performed using SYBR® Premix Ex Taq™IIkit (Takara) in a quantitative PCR System (Bio-Rad, Hercules, CA). The human gene expressions in MSCs in vivo were evaluated using human-specific primers. β-actin was used as an internal reference in all applications. cDNA synthesis was carried out at 50 °C for 30 m, and the PCR conditions were as follows: 30 cycles of denaturaton at 94 °C for 1 m, annaealing at 60 °C for 1 m, and extension at 72 °C for 1 m and one cycle of final extension. The primers sequences used in this study were listed in Supplementary Table S1. All examinations were conducted in triplicate for each cell line.

### Protein isolation and western blot analysis

Total proteins were extracted from the cells by the Radioimmunoprecipitation (RIPA) buffer (25 mM Tris–HCl (pH 7.6), 150 mM NaCl, 1% NP-40, 1% sodium deoxycholate, 1% SDS) supplemented with protease inhibitor cocktail (Roche, Applied Science, Basel, Switzerland), and the concentration of proteins was measured using G250 protein assay (Thermo Fisher Scientific Inc, Shanghai, China) at 595 nm. The samples were separated by 10% Tris-Glycine sodium dodecyl sulfate polyacrylamide gel electrophoresis (SDS-PAGE) gel (Invitrogen) and transferred onto polyvinylidene fluoride (PVDF) membrane (Bio-Rad). The membranes were blocked in 5% BSA for 2 h and then incubated with primary antibodies overnight at 4 °C and secondary antibody for 2 h at room temperature. The bands were visualized by the Western-Light Chemiluminescent Detection System (Pierce Chemical, Rockford, IL). Primary antibodies used in this paper included VEGF, AngII, ERK, p-ERK, LC3, Beclin-1, MEK, Raf, OCN, Sp7, Runx-2, LPL, PPAR, and β-actin and Horseradish peroxidase (HRP)-conjugated secondary antibodies to rabbit and mouse (Santa Cruz Inc, Califomia, USA).

### Enzyme-linked immunosorbent assay (ELISA) of VEGF

The concentration of VEGF was measured as previous paper reported^43,44^. In brief, the different groups of MSCs conditioned medium (CM) were collected as before. Then the CM was concentrated using concentration tube (Milipo) according to the manufacturer’s instructions. After centrifugation (10,000 rpm, 10 m), the concentrated solution was used for analysis. VEGF levels were measured using commercially available ELISA kits (R&D Systems, Minneapolis, USA), following the manufacturer’s protocol. The plates were analyzed by reading the absorbance at 450 nm using Microplate Reader (Bio-Rad). Three independent experiments were performed for each sample.

### Neutralizing VEGF antibody assay

For eliminating the effect of VEGF secreted by MSCs on ECs, we added 5 μl VEGF antibody to the co-culture medium. After incubation at 37 °C for 24 h, the ECs were collected and examined the tube formation.

### LC3 co-immunoprecipitation

Total cell lysates were obtained as detailed above. 200 μl lysates were incubated with rabbit antibody LC3 (MBL International, Woburn, MA, USA) at 4 °C overnight. The control sample were added with IgG. Next day all the samples were added with protein A magnetic beads (Millipore) at 4 °C for further 4 h incubation. For covalent crosslinking, magnetic beads were washed with RIPA buffer (pH = 9.0) for five times. After incubation, the conjugated beads were collected by centrifugation and washed five times with RIPA buffer. Sixty microliters protein lysis buffer was added in beads and boiling at 95 °C for 5 m. The supernatant was collected by centrifugation, immunoprecipitated (IP) proteins and original lysates (input) were separated on 12% SDS-PAGE and immunodetection was performed as described before.

### Flow cytometry analysis

After regulating the level of autophagy, the MSCs were collected and washed twice with PBS and resuspended in 200 μl PBS by repeated vibration to ensure a single-cell suspension. Then the fluorescein-conjugated annexin V and PI was added to cells and incubated for 10 m according to the manufacturer’s protocol. After incubation, the apoptosis analysis of MSCs were detected by flow cytometry (Beckman Coulter).

### Statistical analysis

All data are expressed as mean ± standard deviation (SD) of at least three independent experiments. Differences were performed by One-way ANOVA using the SPSS 17.0 software. A value of *P* < 0.05 was considered to be statistical significant.

## Electronic supplementary material

Autophagy promotes MSC-mediated vascularization in cutaneous wound healing via regulation of VEGF secretion

Supplementary Figure 1

Supplementary Figure 2

Supplementary Figure 3

Supplementary Figure 4

Supplementary Figure 5

Supplementary Figure 6

Supplementary Table S1
