# Supplementary material for: Autophagy promotes MSC-mediated vascularization in cutaneous wound healing via regulation of VEGF secretion
Source: Cell Death Dis. 2018 Jan 19;9(2):58. doi: 10.1038/s41419-017-0082-8 (PMC5833357; doi:10.1038/s41419-017-0082-8)
Supplement: Supplementary file 1 — Autophagy promotes MSC-mediated vascularization in cutaneous wound healing via regulation of VEGF secretion [file 41419_2017_82_MOESM1_ESM.pdf]

Autophagy promotes MSC-mediated vascularization in cutaneous wound healing via regulation of VEGF secretion

An Y[1,2#], Liu WJ[2,3#], Xue P[4#], Ma Y[2], Zhang LQ[2], Zhu B[2,5], Qi M[2],

Li LY[2], Zhang YJ[2], Wang QT [1*], Jin Y [2*]

[1] State Key Laboratory of Military Stomatology & National Clinical Research Center for Oral Diseases & Shaanxi Engineering Research Center for Dental Materials and Advanced Manufacture, Department of Periodontology, School of Stomatology, The Fourth Military Medical University, Xi'an, Shaanxi 710032, China

[2] State Key Laboratory of Military Stomatology & National Clinical Research Center for Oral Diseases & Shaanxi International Joint Research Center for Oral Diseases, Center for Tissue Engineering, School of Stomatology, The Fourth Military Medical University, Xi'an, Shaanxi 710032, China

[3] Xi'an Institute of Tissue Engineering and Regenerative Medicine, Xi'an, Shaanxi 710032, China

[4] Institute of Stomatology, Chinese PLA General Hospital, Beijing 100853, China

[5] Department of Stomatology, PLA Xizang Military Region General Hospital, Lhasa, Tibet 850007, China.

# These authors contributed equally to this work.

*Correspondence: yanjinfmmu@139.com; yanjin@fmmu.edu.cn (Jin Y.),

yznmbk@fmmu.edu.cn (Wang QT.)

Supplementary Figure S1 Isolation and characterization of human mesenchymal stem cells (MSCs). (a) Representative images of single-cell cloning formation from MSCs at day 10. (b) Alizarin red staining of MSCs after 4 weeks of induction in the osteogenic medium (100×). The expression of osteogenesis proteins OCN, SP7 and Runx-2 were detected by western blot. (c) Oil red staining of MSCs after 3 weeks of induction in the adipogenic medium (100×). The expression of adipogenesis proteins LPL and PPAR were detected by western blot. (d) The ultrastructural morphology of autophagosome in MSCs and the protein of Atg7, Beclin-1 and LC3I/II were detected by western blot.

Supplementary Figure S2 Dio-labeled MSCs (red) and LC3-positive cells (green) at 48h after subcutaneous injection. The co-stained cells represented the autophagy activation of MSCs. Scale bar = 50μm.

Supplementary Figure S3 There was no distinction between control group and si-control group. (a) The expression of proteins VEGF, Atg7, Beclin-1 and LC3I/II were detected by western blot. (b) Immunohistochemistry staining on CD31 of wound area tissues 2-week post-operative ($^{NS}P > 0.05$, n=3). Scale bar = 50μm. (c、d) Images of wound size on the back of mouse and quantification of wound healing rate (%). Scale bar = 5mm.

Supplementary Figure S4 Dio-labeled MSCs (red) were detected by immunofluorescence assay 48h after injection. Scale bar = 50μm.

Supplementary Figure S5 Rapamycin had no effect on the wound healing through local application. (a) Images of wound size on the back of mouse and quantification of wound healing rate (%). Scale bar = 5mm. (b) Hematoxylin and eosin (H&E) staining of wound area tissues at 2-week post-operative. The red boxes indicate the skin wound healing. Scale bar = 2mm. (c) Immunohistochemistry staining on CD31 of wound area tissues and quantification of positive area of CD31 at 2-week post-operative ($^{NS}P > 0.05$, n=3). Scale bar = 50μm.

Supplementary Figure S6 VEGF was transfected by siRNA. (a) The protein of VEGF was decreased at 48 hours after transfection (n=3). (b) The gene of VEGF was decreased at 48h after transfection ($^{NS}P > 0.05$, n=6).

Supplement Table S1 Specific primer sequences used for reverse transcriptase-polymerase chain reaction analysis.
