# Supplementary figures and images for: Autophagy promotes MSC-mediated vascularization in cutaneous wound healing via regulation of VEGF secretion
Source: Cell Death Dis. 2018 Jan 19;9(2):58. doi: 10.1038/s41419-017-0082-8 (PMC5833357; doi:10.1038/s41419-017-0082-8)

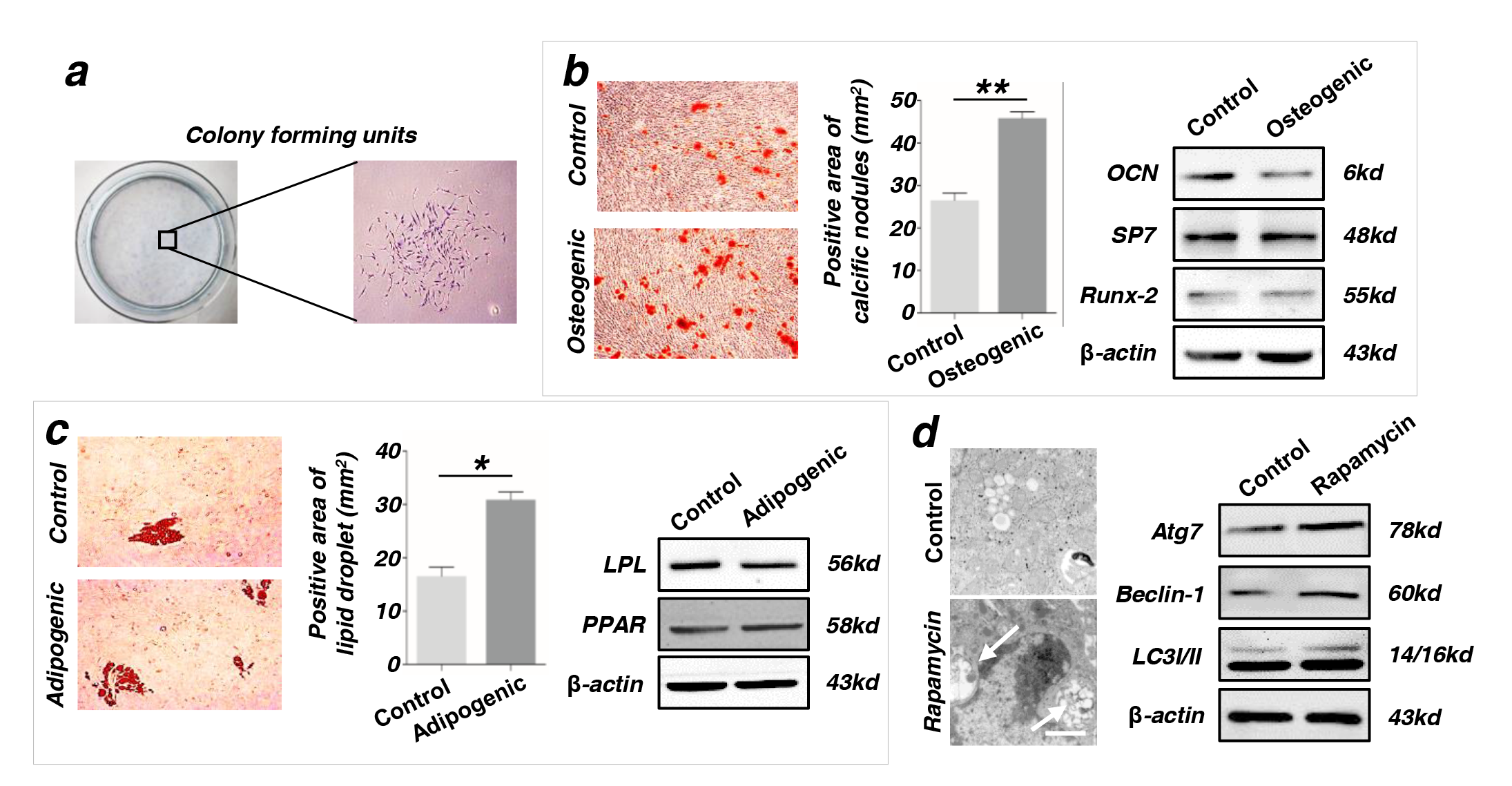

Supplement: Supplementary file 2 — Supplementary Figure 1 [file 41419_2017_82_MOESM2_ESM.tif]

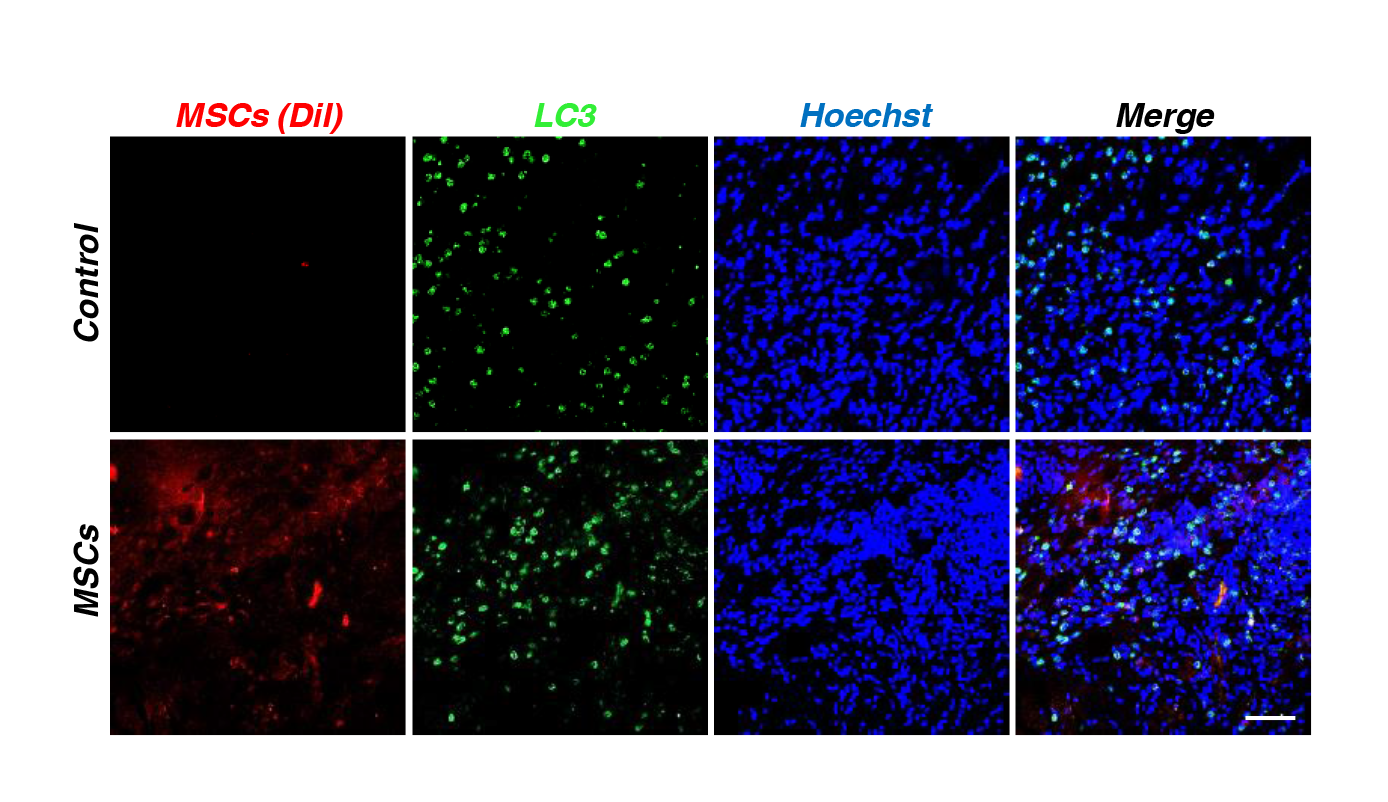

Supplement: Supplementary file 3 — Supplementary Figure 2 [file 41419_2017_82_MOESM3_ESM.tif]

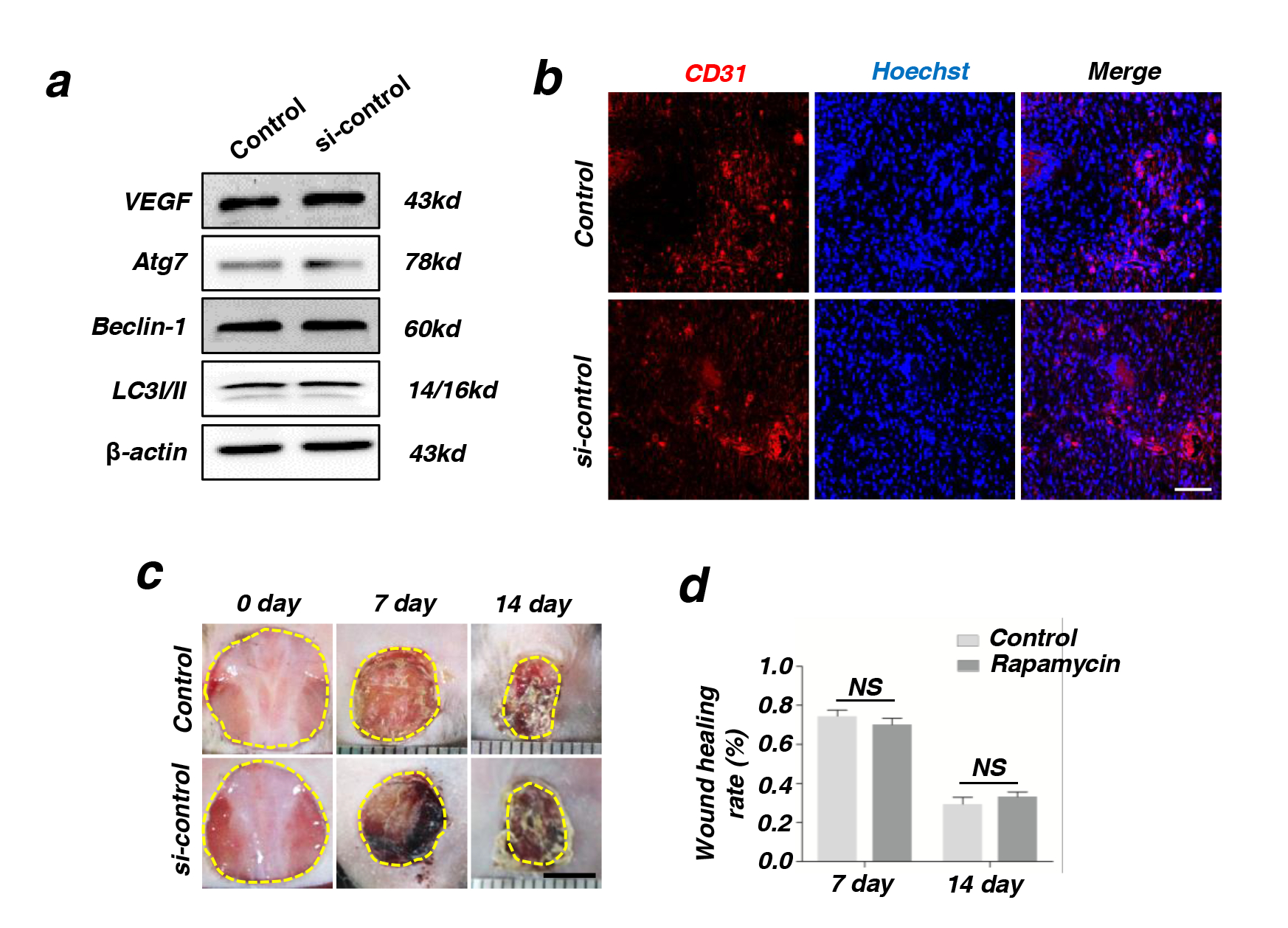

Supplement: Supplementary file 4 — Supplementary Figure 3 [file 41419_2017_82_MOESM4_ESM.tif]

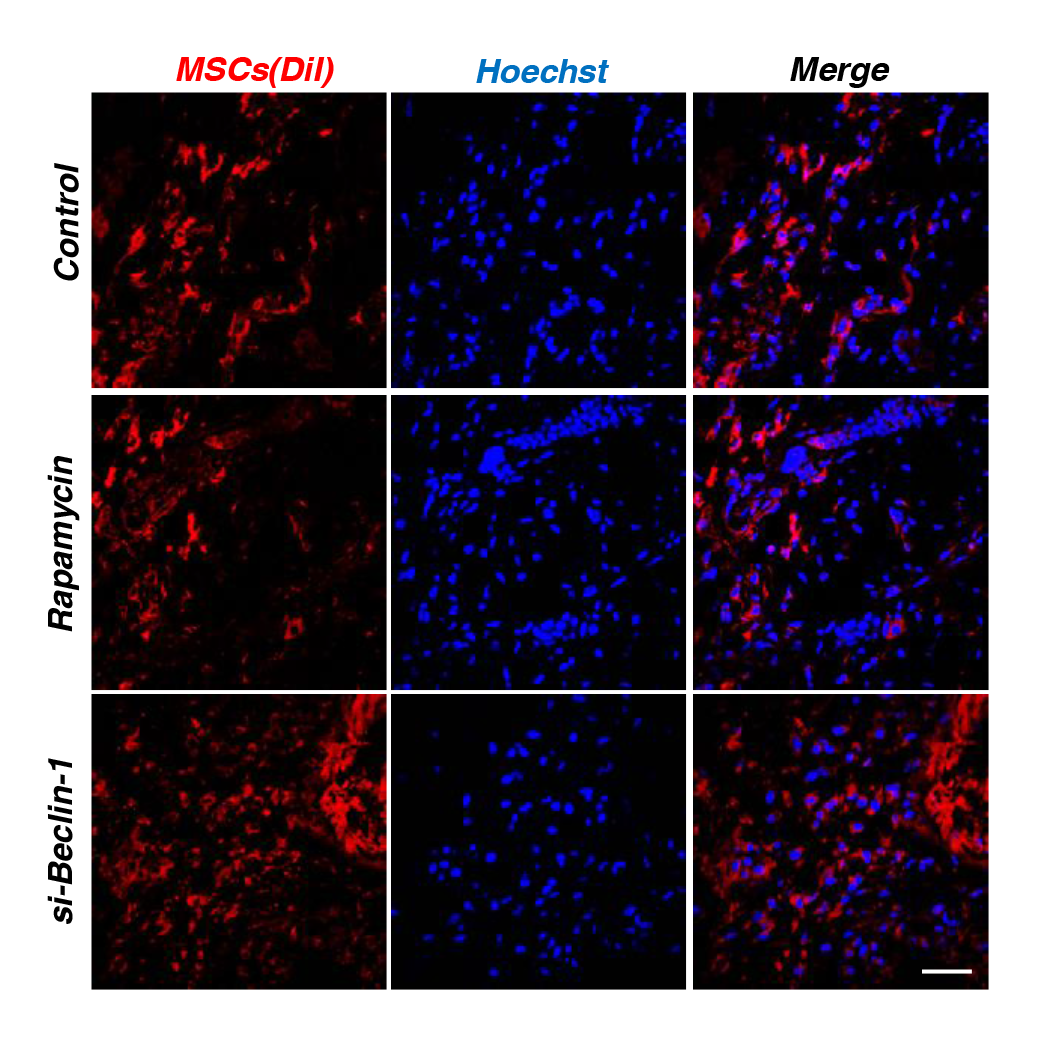

Supplement: Supplementary file 5 — Supplementary Figure 4 [file 41419_2017_82_MOESM5_ESM.tif]

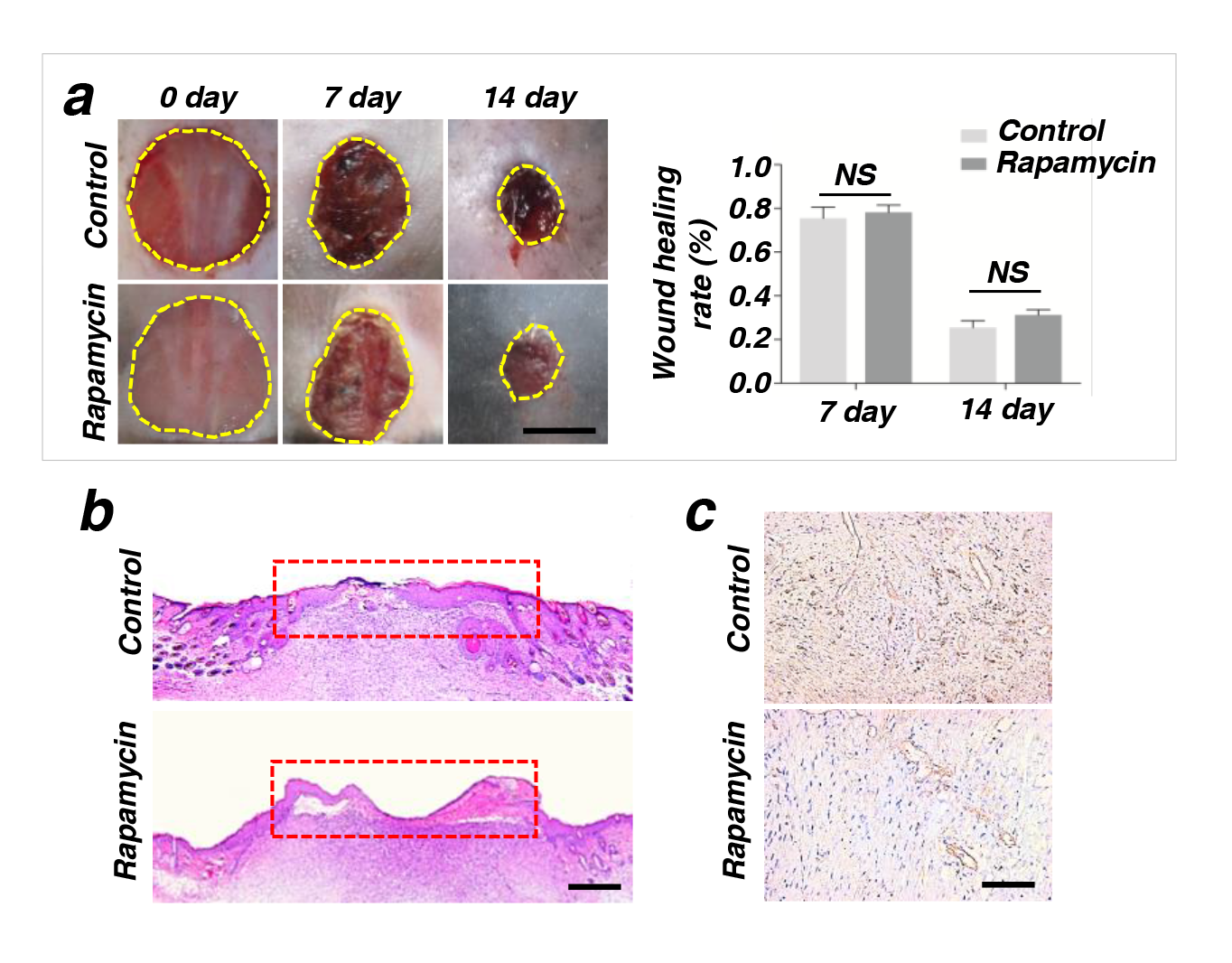

Supplement: Supplementary file 6 — Supplementary Figure 5 [file 41419_2017_82_MOESM6_ESM.tif]

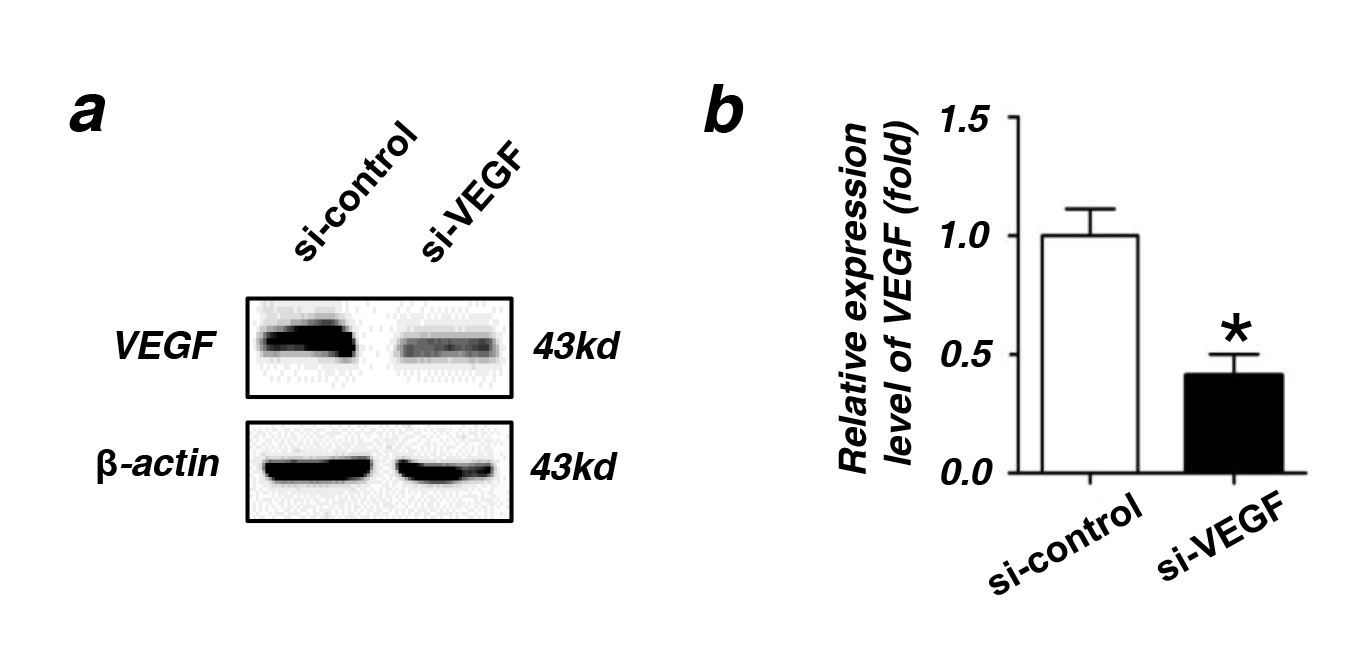

Supplement: Supplementary file 7 — Supplementary Figure 6 [file 41419_2017_82_MOESM7_ESM.tif]
