# Supplementary material for: Autophagy promotes MSC-mediated vascularization in cutaneous wound healing via regulation of VEGF secretion
Source: Cell Death Dis. 2018 Jan 19;9(2):58. doi: 10.1038/s41419-017-0082-8 (PMC5833357; doi:10.1038/s41419-017-0082-8)
Supplement: Supplementary file 8 — Supplementary Table S1 [file 41419_2017_82_MOESM8_ESM.docx]

**Supplementary Table S1**

Specific primer sequences used for reverse transcriptase-polymerase chain reaction analysis.

| Abbreviations | Forward sequences (5’-3’) | Reverse sequence (5’-3’) |
| --- | --- | --- |
| VEGF | TCGAGTCCGAGGGGGCCCAA | GAGCCAGGTCTCCCCGGCGTT |
| PCNA | GGCCAGAGCTCTTCCCTTAC | GTCCTTGAGTGCCTCCAACA |
| FGF | CTCTACTGCAAGAACGGCGG | TGTAACACACTTAGAAGCCAGCA |
| AngII | TGGAACCCATCTCCCGTTGA | AGACCAACAACAAAACGCCC |
| PDGF | AGGCCTGAGCGCCTGAT | TCGAGTGGTCACTCAGCATC |
| TGF | CACGTGGAGCTGTACCAGAA | CCGGTAGTGAACCCGTTGAT |
| Beclin-1 | ACACATTTGTTTGCTGCGGA | TGTGTAAACAAACGACGCCT |
| LC3 | GGGGCCTCGGAGCAAGTCCA | CCCCGGGAGCCTCGTTCAGGT |
| Atg7 | GGATATGGATATTAATTTCC | CCTATACCTATAATTAAAGG |
| β-actin | GCACCGTCAAGGCTGAGAAC | TGGTGAAGACGCCAGTGGAG |
